# Application of *Enterococcus malodoratus* SJC25 for the Manufacture of Whey-Based Beverage Naturally Enriched with GABA

**DOI:** 10.3390/foods11030447

**Published:** 2022-02-02

**Authors:** Daniela S. Cunha, Márcia C. Coelho, Susana C. Ribeiro, Celia C. G. Silva

**Affiliations:** Institute of Agricultural and Environmental Research and Technology (IITAA), University of the Azores, 9700-Angra do Heroísmo, Portugal; daniela.sv.cunha@gmail.com (D.S.C.); marciacoelho8282@gmail.com (M.C.C.); susana.ic.ribeiro@uac.pt (S.C.R.)

**Keywords:** GABA, whey, milk, LAB, dairy, probiotics, glutamic acid, cheese, fermentation

## Abstract

Gamma-aminobutyric acid (GABA) is used as a dietary supplement because of its health-promoting properties. However, concern over the use of synthetic products has increased the demand for foods that are naturally fortified with GABA. In addition, excess whey is a major concern for the dairy industry due to the high cost of treating it. Here, we report the use of a novel *Enterococcus malodoratus* strain isolated from cheese to produce sweet whey beverages naturally enriched with GABA. After the screening of cheese isolates, *E. malodoratus* strains were identified as high GABA producers. One beverage was prepared from pasteurized sweet whey enriched in glutamic acid and *E. malodoratus* SJC25. The fermented beverages were supplemented with a fruit preparation and subjected to chemical, microbiological and sensory analysis. The bacterial counts and GABA content were maintained until storage at 4 °C for 14 days. High conversion rates of glutamic acid to GABA (50–71%) were obtained in the beverages. The GABA content in whey-based beverages reached 250–300 mg/100 mL, which is equivalent to the content of commercially available GABA supplements. The beverages received a positive rating (4/5) by the taste panel. To our knowledge, this is the first report on *E. malodoratus* as a potential GABA producer.

## 1. Introduction

Motivated by consumer health consciousness, the food industry has prompted researchers to develop a greater variety of healthy products with the addition of probiotics and bioactive compounds [1]. Commercially available probiotics mainly include strains of *Lactobacillus* spp. and *Bifidobacterium* spp. used in fermented dairy products [2,3]. In addition, other bacterial species such as *Streptococcus* spp., *Bacillus* spp. and *Enterococcus* spp. are widely used as probiotics in foods and dietary supplements due to their high survival rate in the gut and associated high biological activities [2,4,5,6,7].

Currently, the most internationally accepted definition of a probiotic comes from the Food and Agriculture Organization of the United Nations/World Health Organization, which defines probiotics as “live microorganisms administered in sufficient quantity to provide a health benefit to the host” [8]. From a technological point of view, probiotic microorganisms must allow large-scale production, withstand processing, have a stable acidity, and have an appropriate taste and pleasant texture after fermentation. They must also maintain a high number of viable cells during storage [9].

Several studies have reported the ability of probiotics to alleviate psychological stress in humans and to exert anxiolytic effects in rats, contributing to a growing body of evidence that the gut microbiota can influence the nervous system [10,11,12]. Gamma-aminobutyric acid (GABA) is the main inhibitory neurotransmitter in the central nervous system and has been associated with beneficial health effects such as relaxing and calming effects, with a particular emphasis on the antihypertensive effect demonstrated in animal and human studies [13]. Studies have also demonstrated the ability of various lactic acid bacteria (LAB) to produce GABA, belonging to *Lactococcus*, *Lactobacillus* and *Streptococcus* [7,14]. The production of this neurotransmitter by using LAB in fermented foods is a preferred alternative to satisfy the demand for naturally GABA-enriched products [15]. A still unclear issue is the amount and frequency of consumption of probiotics necessary to ensure the functional benefits. Although, some authors recommend a minimum daily intake of 100 g of fermented dairy products containing 10^7^ CFU/mL [16].

Whey is the watery part of milk which separates from the curds in the manufacture of cheese. A distinction is made between sweet whey, which is obtained by the addition of rennet (pH = 6–7), and acid whey, when coagulation occurs through the action of acid (pH < 5) [17]. This by-product contains about 50% of the nutrients/dry matter of milk [18], including lactose (70–80% DM), proteins (9% DM), minerals (8–20% DM) and other minor components [17,19]. The content of lactose and other nutrients makes whey a potential raw material for the development of probiotic microorganisms that enable the production of fermented beverages [18,20]. As a by-product of cheese production, whey can pollute the environment [21]. The excess of this by-product is one of the major problems for the dairy industry, especially for small and medium enterprises, because the cost of its treatment is high [22]. Dairy beverages fermented from whey and with probiotic cultures added represent an excellent innovative alternative, as they have functional properties and allow the use of whey, minimizing the negative impact on the environment, without requiring large investments or significant changes in the production routine [21]. Studies reporting the development of fermented dairy beverages from whey show high acceptance from a sensory point of view [23,24,25,26].

In previous studies, lactic acid bacteria were isolated from an artisanal cheese made from raw cow’s milk [27]. The aim of the present work was to screen these isolates for converting glutamic acid to GABA and evaluate the use of a GABA producer—*E. malodoratus* SJC25 in the preparation of a drink made from cow’s whey, to which a fruit concentrate was added to improve its sensory properties. To this end, we studied the GABA content and the physicochemical and sensory properties of the fermented whey-based beverages. To our knowledge, this is the first report of the use of *E. malodoratus* as a GABA producer in a flavored beverage made from sweet whey.

## 2. Materials and Methods

### 2.1. Screening of GABA-Producing LAB

A total of 27 strains of lactic acid bacteria (LAB) previously isolated from traditional Azorean cheeses (made from non-pasteurized cow’s milk), were screened for their GABA-producing ability.

The 16S rDNA sequences of isolates have been deposited in the GenBank under accession numbers MT742854–MT742858, MT742859, MT742860, MT742864, MT742869, MT742871, MT742872, MT742878, MT742883, MT742900–MT742907, MT742917–MT742923.

Before being used, the strains were propagated in de Man Rogosa Sharpe (MRS) broth (AES, France) and incubated at 30 °C (non-shaking aerobic incubation), for 48 h. In order to select a LAB with high GABA-producing ability, the optical density (OD) of the inoculum was adjusted to 6.0–6.5 on the McFarland scale. Aliquots of 50 µL of each sample were inoculated into 5 mL of MRS broth, supplemented with 3% glutamic acid (Sigma, Darmstadt, Germany), followed by incubation at 30 °C for 72 h. After incubation, media were centrifuged at 14,000× *g* for 10 min (model 5804R, Eppendorf, Darmstadt, Germany) and supernatants were stored at −17 °C until analysis of GABA and glutamic acid (GLU).

### 2.2. Quantification of GABA and GLU

GABA and GLU contents were determined by HPLC (VWR Hitachi, model Elite Lachrom, Japan) equipped with an automated injector, and a RP-18 column (Purospher^®^ STAR, 5 μm particle size), according to the method described by Li et al. [28], with some modifications. Briefly, samples (100 µL) of culture MRS broth with 3% GLU (supernatants) or fermented beverages were mixed with 900 µL of 20% trichloroacetic acid (TCA, Sigma, Darmstadt, Germany). The mixture was vortexed and centrifuged for 10 min at 4000× *g*. After centrifugation, the supernatant was filtered with 0.45 µm filters (Whatman, Darmstadt, Germany) and used for HPLC analysis. Standards and samples were previously derivatized with *o-*phtaldehyde (OPA). The derivatization reagent (OPA) was prepared with 10 mg of *o*-phtaldehyde (Sigma), 10 µL of 2-mercaptoethanol (99% extra pure, Acros organics, Geel, Belgium) and 2.5 mL of acetonitrile (Sigma), in a tube protected from light. For the derivatization reaction, 100 µL of sample/standard was mixed with 500 µL of borate buffer (0.4 M, pH 10.4) and 100 µL of OPA. The mixture was vortexed for 30 s and reacted at room temperature for 5 min and was immediately injected (20 µL) into the HPLC apparatus. The separation of OPA-derivatives was performed with a mobile phase consisting of ammonium acetate buffer (0.02 M, pH 7.3) as solvent A, and acetonitrile as solvent B. The gradient elution program was set at 80% solvent A and 20% solvent B for 8 min, ramped at 100% of B for 8 min, then at 20% of B until the end of the run (25 min), with a flow rate of 0.6 mL/min. Detection was performed with a Diode Array Detector (DAD), at a wavelength of 334 nm. Calibration curves for GABA and glutamic acid (GLU) were produced using standard standards of GABA (100, 200, 300, 400, 500 and 600 mg/L) and GLU (1, 2.5, and 5 g/L).

### 2.3. Beverage Production

The main raw material used in the formulation of the drink was cow’s sweet whey with an average pH of 6.44, resulting from the production of cured cheese, and was supplied by a cheese producer (Quinta dos Açores, Terceira, Portugal).

The whey was subjected to pasteurization at 90 °C for 5 min. After completing the pasteurization, the whey was cooled in an ice bath until reaching a temperature of 30–32 °C. The beverages were produced with the LAB culture with the greatest potential for producing GABA. The inoculum (5% *E. malodoratus* SJC25 culture, 6.0–6.5 McFarland scale) was centrifuged at 4000× *g* for 10 min, at 10 °C (Eppendorf centrifuge, Model 5804R, Darmstadt, Germany). In order to eliminate all traces of MRS broth, the pellet was washed twice with 20 mL of sterilized water.

Fermentation was performed using sweet whey supplemented with GLU (0.5%) and 5% (*v*/*v*) of GABA-producer (*E. malodoratus* SJC25 culture). After addition of inoculum (5%), fermentation started in an incubator hood (TH 30, Edmund Bühler GmbH, Bodelshausen, Germany) combined with a universal shaker (80 rpm, Edmund Bühler GmbH), at 30 °C for 48 h. The fermented whey was then separated into two sterilized flasks, and 9% of fruit preparation was added to test 2 different formulations: (1) pineapple beverage: whey + GLU (0.5%) + 5% (*v*/*v*) *E. malodoratus* SJC25 + 9% pineapple preparation (water, 30% pineapple puree, sucrose, modified starch, flavors, preservative: potassium sorbate, acidity regulators: citric acid and trisodium citrate and coloring: lutein); (2) passion fruit beverage: whey + GLU (0.5%) + 5% (*v*/*v*) *E. malodoratus* SJC25 + 9% passion fruit preparation (water, 30% passion fruit puree, sucrose, modified starch, acidity regulators: trisodium citrate and citric acid, flavors, preservative: potassium sorbate and coloring: beta carotene). A control formulation was used with whey + GLU (0.5%). Beverages were kept in the refrigerator (4 °C) until being analyzed after 0, 7 and 14 days. Experiments were performed in triplicate.

**Figure 1:**
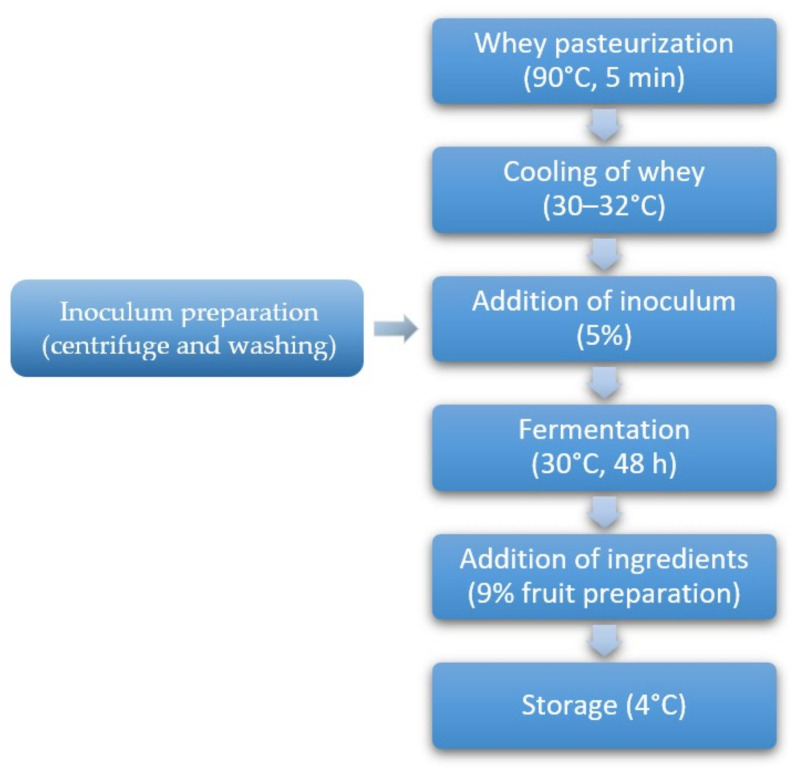
Protocol for the manufacture of the fermented whey beverage with *E. malodoratus* SJC25 culture.

**Figure 2:**
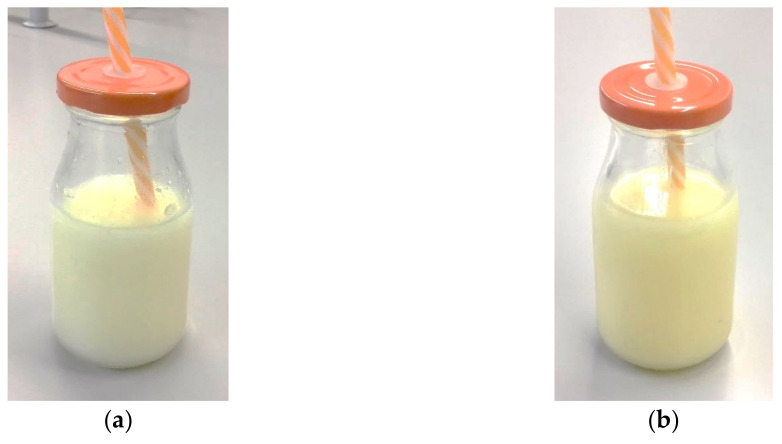
Presentation of the finished product—fermented whey beverage with *E. malodoratus* SJC25. (**a**) Pineapple flavored beverage; (**b**) passion fruit flavored beverage.

The process flowchart is presented in Figure 1 and the images of finished beverages in Figure 2.

### 2.4. Microbiology Analysis

At the end of fermentation, all samples of beverages were serial diluted in peptone water (AES, Rennes, France), plated on MRS (Biokar, Allonne, France), and incubated at 30 °C for 48 h.

Bacterial counts were performed after whey pasteurization, before starting fermentation, at 24 h of fermentation, and in the fermented beverage at 0, 7 and 14 days of storage. Bacterial counts were performed in duplicate.

### 2.5. Chemical Analysis

To evaluate moisture content in the final product, the AOAC method was used [29]. Briefly, 5 g of each sample was dried in the oven at 103 °C for 24 h. Samples were evaluated in quadruplicate.

Titratable acidity was determined by direct titration of 10 mL of each sample, according to the AOAC method [29]. pH was measured directly with a pH meter (WTW Inolab pH Level 1, Weilheim, Germany).

Total sugars were determined according to the method of Dubois et al. [30]. Samples were diluted 1:1000, added (2 mL) to 0.10 mL of phenolic solution and 5 mL of concentrated sulfuric acid. The tubes were vortexed and cooled for 20 min. Absorbance was read on a UV/Vis spectrophotometer (U-2900, Hitachi, Tokyo, Japan) at 490 nm. A calibration curve was constructed with the following glucose concentrations: 0, 12.5, 25, 37.5 and 50 μg/mL. Analyses were performed in triplicate.

Fat content was determined by the Gerber method [29]. In a butyrometer, 10 mL of sulphuric acid was added to 11 mL of the beverage and 1 mL of amyl alcohol, followed by mixing. The butyrometers were centrifuged in the Gerber centrifuge for 5 min at 1200 rpm. Then, they were placed in a water bath at 65 °C for 5 min, and the fat percentage was measured directly in the butyrometer.

Total protein content was calculated from the amount of nitrogen determined by the Micro Kjeldahl method [29]. Nitrogen was converted into protein via multiplication by a factor of 6.38. Briefly, 0.5 g of each sample was digested with sulphuric acid, followed by a distillation with NaOH in 2% boric acid (*w*/*v*). Finally, titration of the obtained distillate was carried out by using 0.1 N HCl.

### 2.6. Sensorial Analysis

Sensory tests were performed to determine the degree of acceptance of the whey-based pineapple and passion fruit beverages. Acceptance tests were carried out on fresh beverages (1–2 days after fermentation) by a panel of 31 untrained adult tasters, usual consumers of dairy products, with a mean age of 37.0 ± 13.2 years (58% women and 42% men). The attributes judged were flavor, aroma, texture and global appreciation. A hedonic scale was used from 1 to 5 points, where 5 represented “Very pleasant/I like it very much” and 1 “Very unpleasant/I do not like anything”. Grades 1 and 2 represented indicators of non-acceptance of beverages. The evaluation regarding the degree of intensity of the attributes acidity and sweetness were evaluated using a scale from 1 to 5 points, where 5 represented “Very intense” and 1 “Slightly intense”.

### 2.7. Statistical Analysis

The analyses were conducted in 3 replicates and presented as mean ± standard error of the mean (SEM). Significant differences between GABA content, pH and bacterial counts throughout storage of beverages were evaluated by one-way analysis of variance (ANOVA) using IBM SPSS Statistics, version 25 (IBM Corporation, New York, NY, USA).

## 3. Results

### 3.1. Screening of GABA-Producing LAB

The results of GABA production in a culture medium enriched with GLU and the respective conversion rate are shown in Table 1. The screening of 27 isolates for the production of GABA included *Enterococcus faecalis*, *E. gilvus*, *E. malodoratus*, *E. devriesei*, *Lacticaseibacillus casei* (former *Lactobacillus casei*), *Lacticaseibacillus paracasei* (former *Lactobacillus paracasei*) and *Leuconostoc mesenteroides*.

In the present study, GABA production ranged from 0 to 13 g/L, which corresponded to a conversion rate between 0 and 54.7%. Of the 27 LAB isolates studied, three clearly stood out for their high potential for GABA production. The isolates identified as *E. malodoratus* SJC24, SJC25 and SJC26 achieved a GABA concentration in the culture medium of 11.0, 13.1 and 12.8 g/L, respectively. Isolate SJC25 produced the highest amount of GABA, with a conversion rate of 43.5%. Therefore, this isolate was selected as inoculum for further fermentation of milk beverages.

### 3.2. Production of GABA in Fermented Beverages

**Figure 3:**
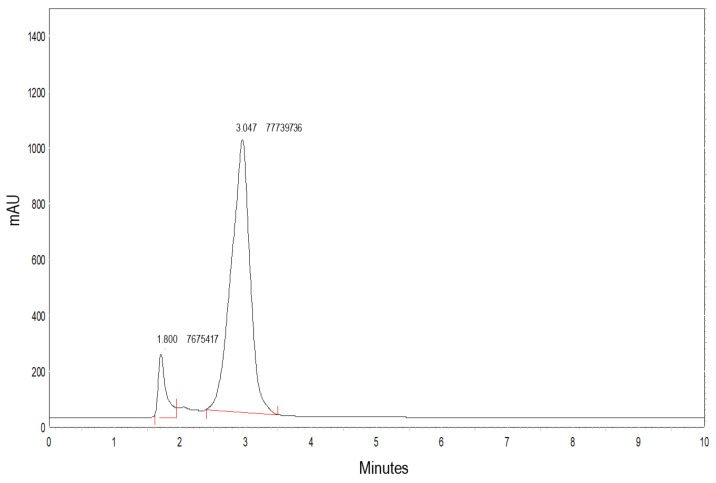
Example of a chromatogram obtained from passion fruit flavor of fermented whey beverage with *E. malodoratus* SJC25, after 14 days of storage at 4 °C.

An example of a chromatogram of a passion fruit whey-based beverage with 14 days of storage at 4 °C is shown in Figure 3. The first peak corresponds to GLU with a retention time of 1.8 min and the second peak corresponds to GABA with a retention time of 3.1 min.

The GABA concentrations obtained in the passion fruit and pineapple beverages are shown in Table 2. The control was the whey without free GLU or GABA. In the whey fortified with 0.5% GLU, a concentration of 4.62 g/L was obtained (recovery rate of 92.4 %).

In both the fermented pineapple and passion fruit beverages, the conversion rates obtained were very high (50–71%).

In the fermented pineapple drink, the original GABA content (2.699 g/L) was maintained for 14 days at 4 °C. GABA content in both pineapple and passion fruit beverages was not significantly different (*p* > 0.05) between different days. In both beverages, residual amounts of GLU were detected in the final product due to the high conversion rates to GABA.

### 3.3. Physico-Chemical and Microbiological Analysis of Beverages

The whey (sweet whey) used in the formulation of the beverages had an initial pH of 6.44, but before fermentation the pH was lowered to 4.35 ± 0.02 as a result of the addition of GLU. At the end of fermentation (after 48 h), the pH of the whey was 4.27 ± 0.01 (data not shown). The pineapple and passion fruit preparations added to the fermented whey also had a low pH (3.8 ± 0.2).

The results of pH, titratable acidity and chemical analyses of the fermented beverages (pineapple and passion fruit) are summarized in Table 3.

Microbiological analyses were performed on the whey after pasteurization (control) and resulted in zero counts in MRS agar. After addition of the inoculum (*E. malodoratus* SJC25) to the whey and before the start of fermentation, the bacterial count was 6.10 ± 0.07 Log CFU/mL. At the end of fermentation (48 h), the average count of LAB was 6.24 ± 0.19 Log CFU/mL (data not shown).

LAB counts in the beverages stored at 4 °C for 14 days are shown in Table 4. During the 14-day storage period, the LAB count remained relatively constant at approximately 6 Log CFU/mL. There were no statistically significant differences (*p* > 0.05) between different days in pH values and bacterial counts. In the pasteurized whey without the addition of the inoculum, LAB was not detected until the 14-day storage at 4 °C.

### 3.4. Sensorial Analysis of Beverages

**Figure 4:**
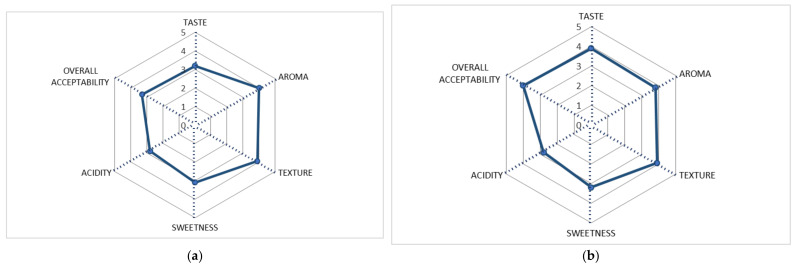
Sensory evaluation of beverages (scores 1–5) of fermented whey beverages: (**a**) pineapple beverage; (**b**) passion fruit beverage.

The results of the 5-level hedonic scale used to evaluate the fermented beverages of pineapple and passion fruit are shown in Figure 4. The pineapple beverage had a mean global rating of 3.3 ± 0.9 and the passion fruit drink of 3.9 ± 0.8, the latter being the drink with the highest acceptability among tasters. Both drinks had a positive acceptability score of above 3.

For the pineapple beverage, aroma and texture were the attributes most accepted by tasters with scores of 3.9 ± 0.9 and 3.8 ± 0.9, respectively, followed by flavor with a score of 3.2 ± 0.9. On average, tasters rated intensity as 2.7 ± 0.9 for acidity and 3.0 ± 1.0 for sweetness.

For the passion fruit beverage, the acceptability scores for the texture, flavor and aroma attributes were relatively close, with scores of 3.9 ± 0.8, 3.8 ± 0.8 and 3.7 ± 0.9, respectively. The tasters estimated the average intensity of 2.7 ± 1.0 for acidity and 3.3 ± 0.8 for sweetness.

## 4. Discussion

The results of the screening for bacterial strains with the ability to produce GABA confirm data reported in several studies in which the ability of LAB to produce GABA varied according to species and strain [15,31,32,33,34]. In addition, several studies suggest that LAB strains isolated from dairy products may have greater potential for GABA production than strains isolated from non-dairy products [33,35].

Of the 27 LABs studied, *E. malodoratus* (strains SJC24, SJC25 and SJC26) stands out as having a high potential for GABA production (11–13 g/L), with conversion rates in the culture medium of approximately 40%. These values are higher than those of several studies that found lower GABA levels (1–6 g/L) in MRS broth enriched with monosodium glutamate (MSG) and under identical fermentation conditions (30 °C, 48 h) [36,37,38]. As far as we know, this is the first study describing this species as a producer of GABA. Most studies investigating GABA-producing LABs have been conducted with the genera *Lactobacillus, Lactococcus* and *Streptococcus*, and only a few studies have been conducted with other genera such as *Enterococcus* [32]. According to some authors, *Enterococcus* spp. are poor producers of GABA [38,39,40]. However, Lee et al. [32] reported an *E. avium* strain that was able to produce high amounts of GABA (13.9 g/L). High GABA production was also attributed to strains of *L. brevis* and *L. buchneri* isolated from kimchi using MRS broth with 5% MSG and pH 5.0 and incubated at 37 °C for 48 h [41,42]. In the present study, strains of *E. malodoratus* achieve comparable levels of GABA production in MRS broth and are also considered high GABA producers. However, there are differences related to the fermentation conditions and the composition of the medium, such as the initial amounts of glutamate or MSG, which may be a determining factor for the GABA concentrations obtained.

In both the fermented pineapple and passion fruit beverages, the conversion rates of glutamate to GABA were very high (50−71%) and higher than the conversion rates obtained in MRS broth culture medium (maximum conversion of 43.5% for LAB SJC25). This result suggests that whey beverages are more favorable matrices for the production of GABA. A relatively small amount of GLU (5 g/L) was initially added to the whey before fermentation to reduce the amount of GLU in the beverage. However, the amount of glutamate remaining in the beverages after fermentation was significantly reduced (to 4–9% of the initially added amount) and reached negligible levels (20–40 mg/100 mL) in the final product. This is noteworthy because high levels of GLU or MSG added to foods have raised some health questions and their safety has caused concern among both researchers and consumers [43,44]. Moreover, the high GABA levels reached at the end of fermentation (2.6–3.1 g/L) do not decrease when stored at 4 °C for 14 days, suggesting that the GABA in the beverage is not converted into other metabolites. This GABA content may have beneficial effects on blood pressure control, among other potential effects [13,14]. For example, daily intake of 10 mg GABA for 12 weeks was shown to be effective in individuals with mild hypertension [45].

Similar levels to the present work (2.7 g/L GABA) were obtained in skim milk fermented with *Lactococcus lactis* ssp. *lactis* isolated from a cheese starter [46]. Other researchers prepared a yogurt with the GABA-producing *S. thermophilus* strain and obtained a similar concentration of GABA (2 mg/mL) in the final product [7]. Similar conversion rates of GLU to GABA were also obtained in beverages prepared with different ingredients, such as brown rice juice, germinated soy juice and skim milk fermented with *Lactococcus lactis* subsp. *lactis* [47], boiled black soy milk fermented with *L. brevis* [14], red seaweed beverage fermented with *L. plantarum* and 1% MSG [48], adzuki bean milk fermented with *L. rhamnosus* [49], lychee juice fermented with *L. plantarum* [50] and sprouted oat flour fermented with *L. plantarum* [51].

In the present study, the acidity and pH values obtained for both beverages are in accordance with the expected acidity values for this type of product [7]. The low pH of whey resulting from the addition of GLU provides favorable conditions for enhanced production of GABA, as the acidity has been shown to promote the activity of glutamic acid decarboxylase (GAD) [50]. In addition, rapid acidification at the beginning of storage is desirable because low pH inhibits the growth of pathogenic and spoilage microorganisms. During storage of whey-based beverage, maintenance of low pH is checked, which is beneficial for the properties of the product. Fluctuations in pH may contribute to the development of undesirable sensory and structural properties of the product [7].

According to some authors, the rate of milk acidification is an extremely important technological factor in the production of fermented milk [52]. In the present study, the strain of *E. malodoratus* remained viable during 14 days storage at 4 °C, which could be due to its high acid tolerance (data not show). Viable counts of *E. malodoratus* SJC25 remained relatively constant (approximately 6 Log CFU/mL) throughout the refrigerated storage. Although there is no general consensus on the recommended levels of probiotics to achieve beneficial effects on host health, it has been suggested that the viability of probiotics should be maintained at 10^6^–10^7^ CFU/mL until the expiry date [53]. The number of viable cells of *E. malodoratus* at the end of the storage period is consistent with this criterion, suggesting that this beverage can be considered a probiotic food. However, since enterococci do not have QPS status [54], the use of *E. malodoratus* strains as probiotics requires further evaluation of their safety. Previous studies have shown that the strain E. malodoratus SFC25 has neither the presence of virulent genes nor resistance to relevant antibiotics such as vancomycin [27]. 

The results of the present study showed that the chemical composition of the beverages was comparable to that of whey and whey-based beverages [18,19,23,25]. The high moisture values of the whey beverages were expected due to the high percentage of whey used in the formulation of the product and are in line with acceptable values for liquid yogurts (maximum 91.5% moisture). The average protein content was also similar to whey [55]. Whey proteins have been reported to have a variety of beneficial effects, both nutritionally and on health [21]. In terms of total sugar content, these levels are slightly lower than many liquid yogurts [56]. Both beverages had a fat content comparable to semi-skimmed yogurt, which has a fat content of about 1.4% [55].

Consumer perception is a critical factor in the development of novel foods, as it influences consumers’ willingness to purchase them. In this regard, acidity has a great impact on the quality characteristics of fermented dairy products and is one of the factors limiting their acceptance. Although the fermentation time was prolonged, there were no undesirable changes in the whey beverage. *E. malodoratus* had no negative effect on the aroma of the beverages, as a mean of 4 was reported for the passion fruit beverage on the five-point hedonic scale. Despite reports attributing to this species the production of H_2_S, which causes an unpleasant odor [57], this characteristic was not observed at all in the beverages produced.

## 5. Conclusions

As far as we know, this is the first work describing *E. malodoratus* as a potential producer of GABA, and the high conversion rates obtained should be emphasized. The GABA concentrations obtained in the produced beverages are higher or equivalent to the amounts reported in the literature, which are necessary to have beneficial effects on consumer health, especially in the control of hypertension. From the results of the present study, it can be concluded that a daily intake of 100 mL of these fermented whey-based beverages would provide a total of 250–300 mg of GABA. 

Whey proved to be a favorable matrix for the production of GABA, which when combined with fruit preparations resulted in a dairy beverage with satisfactory sensory properties (evaluated by the selected panel), suggesting that its integration into the daily eating habits of consumers may be feasible. The beverages developed in this work are an excellent alternative for the use of whey, as it is possible to include in their formulation a large proportion of whey in the liquid phase with good sensory acceptability.

## Figures and Tables

**Table 1:** Screening of lactic acid bacteria (LAB) for production of ɣ-aminobutyric acid (GABA) in MRS broth after 48 h. Values of GABA concentration (mg/L) and percentage of conversion (calculated as the percentage of glutamic acid added to medium converted to GABA) are indicated.

Species Identification	Isolate	Accession Number	GABA (mg/L)	Conversion (%)
*Enterococcus faecalis*	SJC20	MT742854	1787	6.0
*Enterococcus gilvus*	SJC21	MT742855	0	0
*Enterococcus gilvus*	SJC22	MT742856	1403	4.7
*Enterococcus malodoratus*	SJC24	MT742858	11021	36.7
*Enterococcus malodoratus*	SJC25	MT742859	13062	43.5
*Enterococcus malodoratus*	SJC26	MT742860	12823	42.7
*Enterococcus faecalis*	SJC30	MT742864	208	0.7
*Enterococcus devriesei*	SJC35	MT742869	678	2.3
*Enterococcus faecalis*	SJC37	MT742871	0	0
*Enterococcus faecalis*	SJC38	MT742872	16	0.1
*Enterococcus faecalis*	SJC44	MT742878	0	0
*Enterococcus faecalis*	SJC49	MT742883	0	0
*Lacticaseibacillus casei*	SJC66	MT742900	2391	8.0
*Enterococcus faecalis*	SJC67	MT742901	818	2.7
*Leuconostoc mesenteroides*	SJC68	MT742902	318	1.1
*Enterococcus faecalis*	SJC69	MT742903	2477	8.3
*Enterococcus faecalis*	SJC70	MT742904	1278	4.3
*Leuconostoc mesenteroides*	SJC71	MT742905	1269	4.2
*Leuconostoc mesenteroides*	SJC72	MT742906	0	0
*Enterococcus faecalis*	SJC73	MT742907	2667	8.9
*Enterococcus faecalis*	SJC83	MT742917	2197	7.3
*Lacticaseibacillus paracasei*	SJC84	MT742918	2125	7.1
*Enterococcus faecalis*	SJC85	MT742919	2429	8.1
*Enterococcus faecalis*	SJC86	MT742920	2988	10.0
*Lacticaseibacillus casei*	SJC87	MT742921	2354	7.8
*Lacticaseibacillus paracasei*	SJC88	MT742922	803	2.7
*Lacticaseibacillus paracasei*	SJC89	MT742923	2647	8.8

**Table 2:** GLU and GABA concentration in unfermented whey and fermented whey with *E. malodoratus* SJC25 (pineapple and passion fruit flavor beverages). Data are presented as mean ± SEM.

Sample	Time (Days)	GLU (g/L)	GABA (g/L)
Whey	0	ND *	ND *
Whey + GLU (5 g/L)	0	4.620 ± 0.531	ND *
Whey beverage Pineapple flavor	0	0.433 ± 0.058	2.699 ± 0.230
	7	0.351 ± 0.047	2.319 ± 0.198
	14	0.231 ± 0.031	3.117 ± 0.266
Whey beverage Passion fruit flavor	0	0.310 ± 0.036	3.272 ± 0.198
	7	0.200 ± 0.023	3.178 ± 0.192
	14	0.303 ± 0.035	2.636 ± 0.160

* ND: not detected (under detection limit).

**Table 3:** Proximate composition of fermented whey beverages. Samples were analyzed after fermentation and are presented as the average of two samples ± SEM.

	Pineapple Beverage	Passion Fruit Beverage
Titratable acidity (g LA/100 mL)	0.63 ± 0.07	0.73 ± 0.06
pH	4.30 ± 0.04	4.28 ± 0.03
Moisture (g/100 mL)	89.8 ± 0.04	90.2 ± 0.01
Total sugar (g/100 mL)	8.7 ± 1.1	6.5 ± 0.82
Total fat (g/100 mL)	1.0 ± 0.2	1.0 ± 0.2
Protein (g/100 mL)	0.535 ± 0.023	0.513 ± 0.022

**Table 4:** Total LAB counts (Log CFU/mL) on pasteurized whey without addition of inoculum and whey beverages with addition of *E. malodoratus* SJC25, for 14 days of storage at 4 °C. Values are the average of duplicates ± SEM.

Time (Days)	Whey * Log UFC/mL	Pineapple Beverage		Passion Fruit Beverage	
		pH	Log CFU/mL	pH	Log CFU/mL
0	ND	4.30 ± 0.04	6.20 ± 0.10	4.28 ± 0.03	6.30 ± 0.15
7	ND	4.29 ± 0.06	6.34 ± 0.15	4.23 ± 0.03	7.38 ± 0.67
14	ND	4.27 ± 0.06	6.26 ± 0.04	4.17 ± 0.00	6.41 ± 0.12

* ND: not detected.
